# Heritable Multiplex Genetic Engineering in Rats Using CRISPR/Cas9

**DOI:** 10.1371/journal.pone.0089413

**Published:** 2014-03-05

**Authors:** Yuanwu Ma, Bin Shen, Xu Zhang, Yingdong Lu, Wei Chen, Jing Ma, Xingxu Huang, Lianfeng Zhang

**Affiliations:** 1 Key Laboratory of Human Disease Comparative Medicine, Ministry of Health, Institute of Laboratory Animal Science, Chinese Academy of Medical Sciences, Beijing, China; 2 MOE Key Laboratory of Model Animal for Disease Study, Model Animal Research Center of Nanjing University, Nanjing Biomedical Research Institute, National Resource Center for Mutant Mice, Nanjing, China; Mayo Clinic, United States of America

## Abstract

The CRISPR/Cas9 system has been proven to be an efficient gene-editing tool for genome modification of cells and organisms. Multiplex genetic engineering in rat holds a bright future for the study of complex disease. Here, we show that this system enables the simultaneous disruption of four genes (*ApoE*, *B2m*, *Prf1*, and *Prkdc*) in rats in one-step, by co-injection of Cas9 mRNA and sgRNAs into fertilized eggs. We further observed the gene modifications are germline transmittable, and confirmed the off-target mutagenesis and mosaicism are rarely detected by comprehensive analysis. Thus, the CRISPR/Cas9 system makes it possible to efficiently and reliably generate gene knock-out rats.

## Introduction

Rats have returned back to the laboratory as a “renaissance animal” and are becoming an indispensable experimental animal model for understanding the human genome through the establishment of biological links between disease phenotypes and genetic networks. Gene targeting via embryonic stem (ES) cells provides a powerful tool for the generation of precise genetic alterations, but genome manipulation in rat ES cells is inefficient and technically challenging [1]–[2]. Other genome-editing technologies, such as zinc-finger nucleases (ZFNs) [3]–[4] and transcription activator-like effector nucleases (TALENs) [5]–[7] have proven effective for genomic manipulation but are limited because of the need to engineer specific protein pairs for each target site. Recently, the Clustered Regularly Interspaced Short Palindromic Repeats (CRISPR)/CRISPR-associated 9 (Cas9) system has been proven to be a simpler way to edit the eukaryotic genome even in a multiplex manner [8]–[14]. A small single-guide RNA (sgRNA) is produced by fusion of the crRNA and tracrRNA sequences, leading to the formation of Cas9 protein-containing ribonucleoprotein complexes that recognize and cleave specific DNA sequences [8]–[9], [15]. The Cas9 protein and sgRNA are the only components necessary for the induction of targeted DNA cleavage in zebrafish [15], mammal cells [8], [14] and mice [12]–[14]. Furthermore, Cas9 and sgRNA can effectively disrupt multiple genes in cells and different organisms [8], [14]. Here, we extend the application of the CRISPR/Cas9 system to multiplex genetic engineering in the rat.

## Materials and Methods

### Animals

Rats were bred in standard cages in an Assessment and Accreditation Of Laboratory Animal Care-accredited SPF animal facility. All animal protocols were approved by the Animal Care and Use Committees of the Institute of Laboratory Animal Science of Peking Union Medical College (ILAS-GC-2010-044).

### DNA constructs

The paired synthesized oligonucleotides for sgRNAs were annealed and cloned into the pUC57-sgRNA expression vector (Table S1 and Fig. S1 in File S1). The oligonucleotide sequences are listed in Table S1 in File S1.

### *In vitro* transcription

The Cas9 expression plasmid was linearized with *Age* I and used as the template for *in vitro* transcription using the T7 Ultra Kit (Ambion, AM1345) [12]. sgRNA expression plasmids were linearized with *Dra* I and used as templates for *in vitro* transcription using the MEGAshortscript Kit (Ambion, AM1354). Transcribed Cas9 mRNA and sgRNA were both purified by using the MEGAclear Kit (Ambion, AM1908).

### Cas9/sgRNA injection into fertilized rat eggs

Sprague Dawley (SD) rats purchased from Beijing Vital River Laboratories animal center are housed in standard cages and maintained on a 12-h light/dark cycle with food and water. The microinjection of fertilized rat eggs was described previously [3]. In brief, four-week-old donor rats were injected with 30 units of pregnant mare serum gonadotropin (PMSG, Sigma-Aldrich), followed by an injection of 30 units of human chorionic gonadotropin (hCG, Sigma-Aldrich) 48 h later, and immediately mated with SD males. Zygotes were obtained on the next day and cultured in KSOM (Millipore) at 37°C, 5% CO_2_ for 2 h and then prepared for microinjection. In the first experiment, zygotes were injected with a mixture of Cas9 mRNA (20 ng/µl) and sgRNAs containing *ApoE* (10 ng/µl), *B2m* (10 ng/µl), *Prf1* (10 ng/µl), and *Prkdc* (10 ng/µl) to the targeting site A. In the second experiment, zygotes were injected with a mixture of Cas9 mRNA (20 ng/µl) and sgRNAs containing 8 sgRNAs (5 ng/µl each) targeting 2 adjacent sites (targeting site A and B) of each of the 4 genes (Table S2 in File S1). Microinjections were performed in fertilized eggs using a Nikon Microinjection system under standard conditions. Pseudopregnant SD rats were anesthetized by intraperitoneal injections of pentobarbital (Sigma) at 4 mg/100 g of body weight. And then the injected zygotes were transferred to pseudopregnant SD rats (20–30 zygotes per pseudopregnant SD rat) to be carried to parturition.

### T7EN1 cleavage assay

Genomic DNA was extracted from the tails of 7-day-old rats using phenol-chloroform and recovered by alcohol precipitation. The T7EN1 cleavage assay was performed as described by Shen *et al.* [12]. PCR was used to amplify the targeting loci using the following conditions: (95°C, 5 min; [95°C 30 s, 63°C 30 s, 72°C 40 s]×35 cycles; 72°C 10 min; hold at 4°C). PCR products were purified using a PCR clean-up kit (Axygen, AP-PCR-50). Purified PCR products were denatured and re-annealed in NEB Buffer 2 (NEB) prior to digestion with T7EN1 (NEB, M0302L) for 40 min and separation by 2% agarose gel electrophoresis. Cleavage bands in the T7EN1 cleavage assay indicated modification of the targeting site. The corresponding PCR products were sub-cloned for sequencing analysis to detect mutations. The PCR primers used to amplify sgRNA target fragments of *ApoE*, *B2m*, *Prf1* and *Prkdc* are listed in Table S3 in File S1. If no wild type allele was observed by sequencing, we assumed the rat had bi-allelic mutation. If wild type allele was detected by sequencing, we assumed the rat harbored monoallelic mutation. If more than two genotypes were detected by sequencing, we assumed the rat had mosaic mutation.

### Identification and analysis of off-target sites

The following potential off-target loci were searched by BLAST (http://blast.ncbi.nlm.nih.gov/Blast.cgi) using 20 bp sgRNA sequence closed to the PAM (N(G/A)G) and PAM, as NN NNNNN NNNNN A(G/A)G; NN NNNNN NNNNN C(G/A)G; NN NNNNN NNNNN G(G/A)G; and NN NNNNN NNNNN T(G/A)G, where N is the seed base matching the target site exactly [10], [16]–[18]. The *ApoE*, *B2m*, *Prf1*, and *Prkdc* genes were applied to off-target assays and potential target sites (OTS) highly homologous to the target sites summarized in Table S5 in File S1. The selected OTS were amplified from tail genomic DNA and subjected to the T7EN1 cleavage assay and sequencing analysis.

### Germline transmission assay

Germline transmission of the modified genes was determined in the F_1_ rats from crossing an F_0_ rat with a wild-type SD rat. To determine the genotypes of the F_1_ offspring, PCR products of tail genomic DNA from F_1_ rats were subjected to the T7EN1 cleavage assay and sequencing analysis. Detection of mutation from F_1_ rats identical to the mutation from F_0_ parents was considered as germline transmission.

### Biochemical parameters

Fasting blood sample was collected from tail of the rat after hungry for the night. Whole blood was centrifuged at 3000 g for 15 min at room temperature to obtain the serum and prepared for serum total cholesterol (CHO), triglycerides (TG), high density lipoprotein (HDL) and low density lipoprotein (LDL) detection using HITACHI 7100 Automatic Analyzer.

### Flow cytometry analysis

The peripheral blood cells were lysised with BD Pharm Lyse™ Lysing buffer and filtered with a sterile nylon mesh. After counted, cells were stained with B cell marker, Anti-Rat CD45RA APC (eBioscience) and T cell marker Anti-Rat CD3 PE (eBioscience). Data acquisition was performed on FACS Aria I (Becton Dickson) and analyzed on the FlowJo software.

### Western blot analysis

Equal amounts of soluble protein were separated by SDS-PAGE and transferred onto a nitrocellulose membrane (Immobilon NC; Millipore, Molsheim, France). Immunoblotting was carried out with antibodies specific for B2M (1∶1000, proteintech). Primary antibodies were visualized with anti-rabbit HRP-conjugated secondary antibodies (Santa Cruz) using a chemiluminescent detection system (Western blotting Luminal Reagent; Santa Cruz). Variations in sample loading were corrected by normalizing to β-actin levels.

## Results

### Generation of multiple gene modification using one sgRNA for each gene by zygote injection

Four genes, including apolipoprotein E (*ApoE*), beta-2 microglobulin (*B2m*), perforin 1 (*Prf1*), and protein kinase DNA-activated catalytic polypeptide (*Prkdc*), were selected to determine whether the CRISPR/Cas9 system could be used to disrupt multiple genes in rats. The CRISPR/Cas9 targeting sites were designed using the rules described as before [10], [16]–[17]. The Cas9 (Addgene No. 44758) and sgRNAs were transcribed by T7 RNA polymerase *in vitro* as described by Shen *et al.* (Fig. S1 in File S1). Twenty nanogram Cas9 mRNA and sgRNA mixtures were pooled and microinjected into one-cell fertilized eggs of SD rats (Table S2 in File S1).

**Figure 1:**
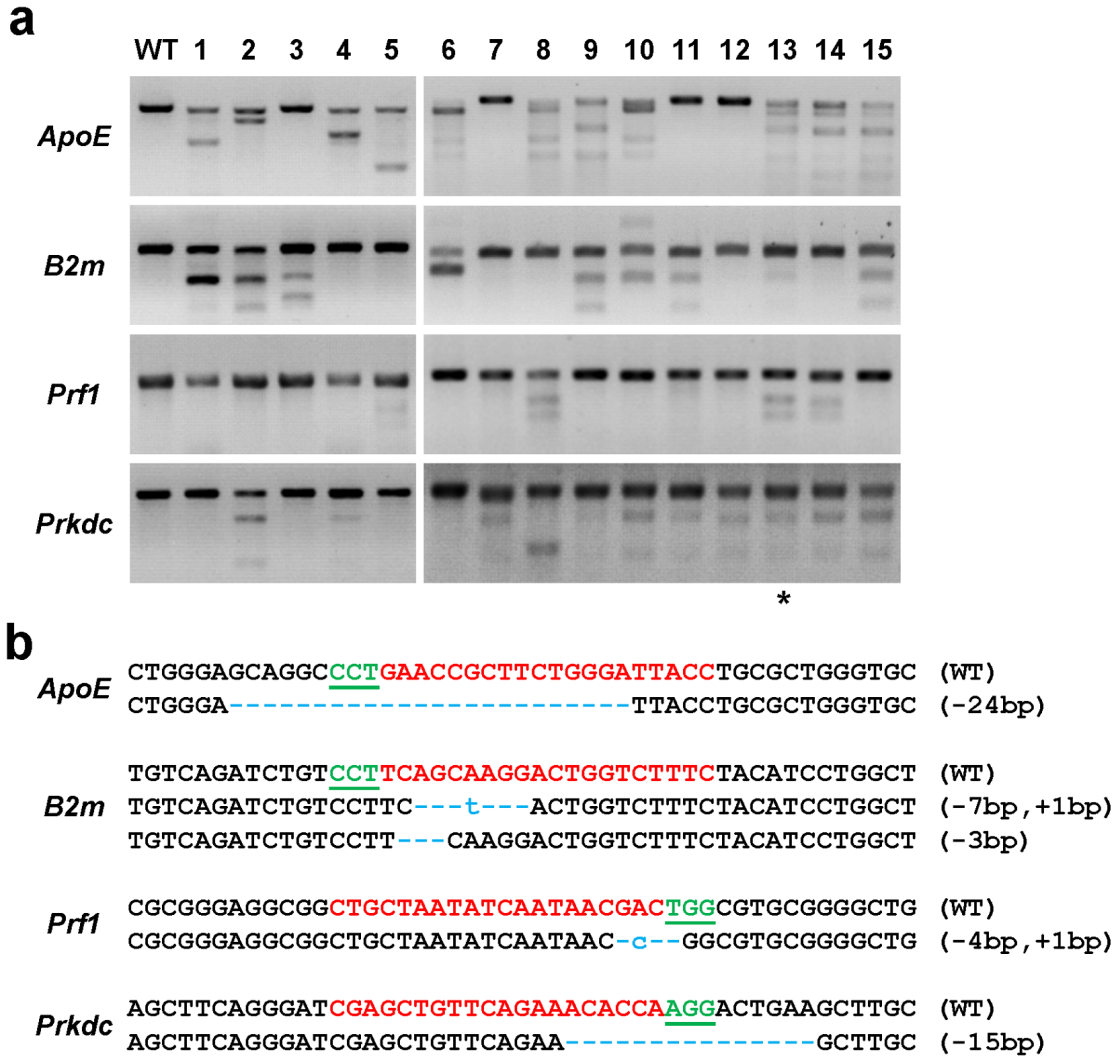
Generation of multiplex genetic modified rats using CRISPR/Cas9 system. (a) Detection of Cas9:sgRNA-mediated site-specific cleavage of the endogenous *ApoE*, *B2m*, *Prf1*, and *Prkdc* by T7EN1 cleavage assay. PCR amplicon of the targeted fragment at the *ApoE*, *B2m*, *Prf1*, and *Prkdc* in 15 founder rats (#1∼15) were subjected to T7EN1 cleavage assay. Founder #13, which is quadruple gene mutant, was marked with asterisks. (b) DNA sequences of four loci in founder #13. PCR amplicon with cleaved bands in T7EN1 cleavage assay were cloned and sequenced. The PAM sequence was underlined and highlighted in green; the targeting site are red; the mutations are blue, lower case; insertions (+) or deletions (−) are shown to the right of each allele.

**Figure 2:**
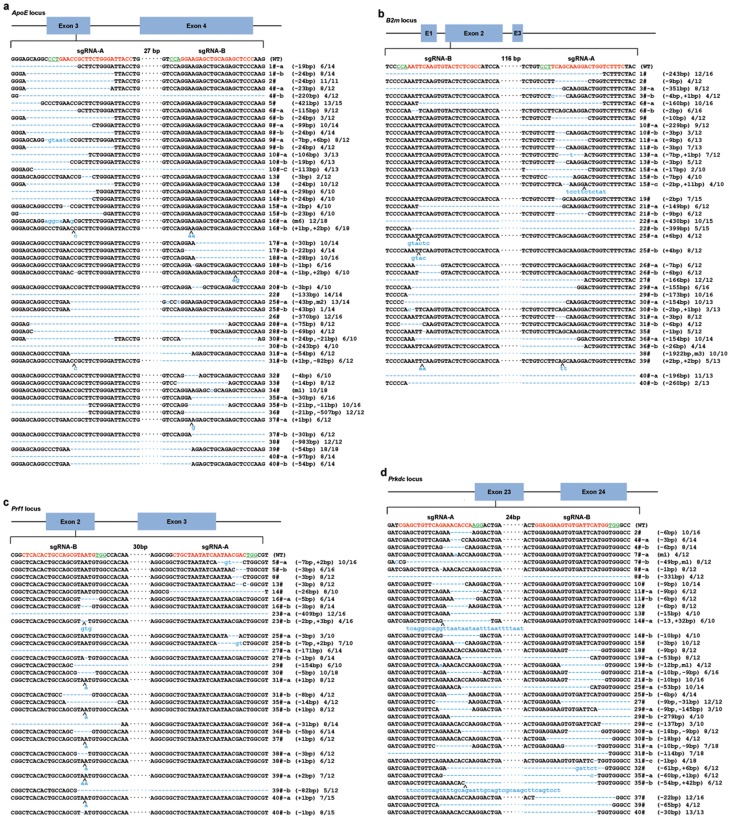
Schematic diagrams of sgRNAs and DNA sequences of targeting genomic loci. PCR amplicon of the targeted fragment in the *ApoE*, *B2m*, *Prf1*, and *Prkdc* in all founder rats (#1∼40) were sequenced. The PAM sequence is underlined and highlighted in green; the targeting site are red; the mutations are blue, lower case; insertions (+), deletions (−) or mutant (m) are shown to the right of each allele. N/N indicates positive colonies out of total sequenced. (a) *ApoE* locus. (b) *B2m* locus. (c) *Prf1* locus. (d) *Prkdc* locus.

A mixture of 4 sgRNAs, each targeting one gene (targeting site A) (Fig. 1b), was used at 10 ng/µl/sgRNA. Total 125 injected zygotes were transferred to 5 pseudopregnant female SD rats, and 15 pups were born (Table S2 in File S1). To detect the gene modifications, the targeted loci were amplified as 500∼700 bp fragments around each targeting site (Fig. S2 and Table S3 in File S1). The PCR products were digested by T7 endonuclease 1 (T7EN1), an enzyme that is capable of recognizing and cleaving mismatched DNA (Fig. 1a). The results showed, the cleavages were detected at all the 4 targeted sites with efficiency of 73.33% at *ApoE* (founders #1, #2, #4∼6, #8∼10, #13∼15), 60% at *B2m* (founders #1∼3, #6, #9∼11, #13, #15), 26.67% at *Prf1* (founder #5, #8, #13, #14), and 66.67% at *Prkdc* (founders #2, #4, #7, #8, #10∼15), respectively (Fig. 1a, Table 1). Further sequencing confirmed the loci were indeed mutagenized in these rats (Fig. 2). Our results indicated the CRISPR-Cas9 function efficiently at all targeted loci. Furthermore, the T7EN1 cleavage assay and sequencing results showed three rats (3/15) contained one mutant gene, six rats (6/15) contained two mutant genes, five rats (5/15) contained three mutant genes, and one rat (1/15) contained four mutant genes (Fig. 1b, Table S4 in File S1). It suggests that Cas9:sgRNA system can disrupt multiple genes in one step.

**Table 1:** Summary of the mutation of the founder rats.

		Mutant rats/Total rats (%)				Bi-allelic mutant rats/Total rats (%)			
	Total	*ApoE*	*B2m*	*Prf1*	*Prkdc*	*ApoE*	*B2m*	*Prf1*	*Prkdc*
Single sgRNA/Gene	15/15 (100%)	11/15 (73.3%)	9/15 (60%)	4/15 (26.7%)	10/15 (66.7%)	10/15 (66.7%)	6/15 (40%)	1/15 (6.7%)	5/15 (33.3%)
Dual sgRNAs/Gene	24/25 (96%)	19/25 (76%)	14/25 (56%)	13/25 (52%)	13/25 (52%)	15/25 (60%)	11/25 (44%)	10/25 (40%)	9/25 (36%)

Single sgRNA/Gene: A mixture of 4 sgRNAs, each targeting a single site in each of the 4 genes. Dual sgRNAs/Gene: Another distinct sgRNA of each gene, together with the tested 4 sgRNAs targeting double site in each of the 4 genes.

### Two sgRNAs were used for multiple genes modification in rats

**Figure 3:**
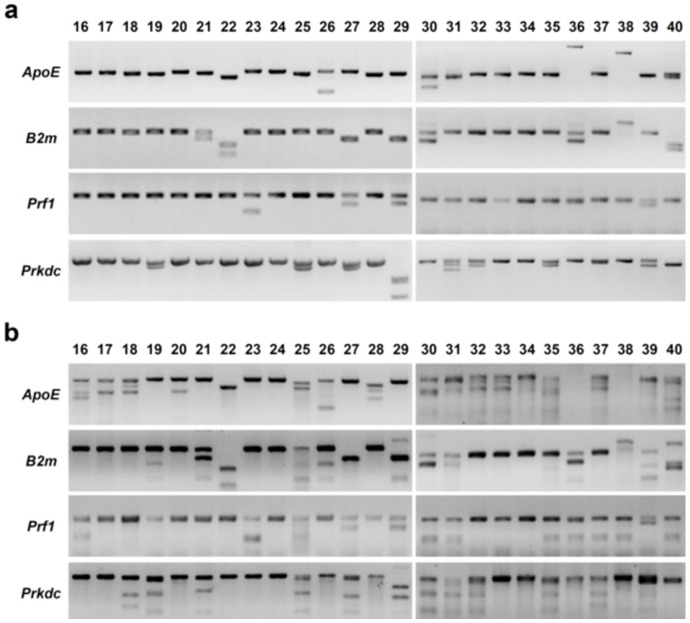
Cas9:sgRNA-mediated modifications in 4 genes by a mixture of dual sgRNAs for each gene. (a) PCR identification of sgRNA:Cas9-mediated site-specific cleavage of the endogenous *ApoE*, *B2m*, *Prf1*, and *Prkdc* loci. The genetic modification analysis was performed by PCR amplification of the targeted fragment in the *ApoE*, *B2m*, *Prf1*, and *Prkdc* in 25 founder rats (#16∼40) derived from co-microinjection of a mixture of dual sgRNAs for each genes as described in Table S2 in File S1. Primer used for PCR amplication was described in Table S3 in File S1. Additionally, founder #36, #38 had a larger deletion in the *ApoE*, the primer *ApoE*-NS2 and *ApoE*-NAS2 used for amplification. Founder #38 had a larger deletion in the *B2m*, the primer *B2m*-S2 and *B2m*-AS2 used for amplification. (b) Detection of Cas9:sgRNA-mediated site-specific cleavage of the endogenous *ApoE*, *B2m*, *Prf1*, and *Prkdc* by T7EN1 cleavage assay. PCR products from (a) were subjected to T7EN1 cleavage assay as described in material and methods.

Considering that two sgRNAs targeting adjacent sites efficiently deleted the intervening region in cells [8], we tested whether using dual sgRNAs targeting at one gene enable fragment deletion by CRISPR/Cas9 system in rats. Therefore, another distinct sgRNA of each gene, together with the tested 4 sgRNAs were co-microinjected into one-cell-stage SD rat embryo at 5 ng/µl/sgRNA (Table S2 in File S1). A total of 26 pups from 9 recipients were born from 276 transferred embryos (one died after birth) (Table S2 in File S1). The modifications of the different loci were also analyzed by PCR (Fig. 3a), T7EN1 cleavage assay (Fig. 3b), and sequencing (Fig. 2). The CRISPR/Cas9 mediated the cleavage of target loci with high efficiencies of 76%, 56%, 52%, and 52% at *ApoE*, *B2m*, *Prf1*, and *Prkdc*, respectively (Table 1). Compared with single sgRNA, dual sgRNAs targeting yielded more fragment deletion from PCR and sequencing results (Fig. S2 in File S1, Fig. 2, Fig. 3a). Notably, almost all the deleted fragments cover the dual sgRNAs targeting sites or locate between the two sites (Fig. 2). Interestingly, targeted *ApoE* locus (in potential founder #36 and #38) and *B2m* locus (in potential founder #38) can't be PCR amplified using normal primers, suggesting a larger fragment was deleted. Indeed, using new primers we confirmed large fragment deletion up to 983 bp in length in the *ApoE* (potential founder #38, Fig. 2 & 3), 1922 bp in length in the *B2m* (potential founder #38, Fig. S2 & S3 in File S1). More importantly, dual sgRNAs targeting yielded 6 rats (6/25) harboring all 4 mutant genes, which is higher than single sgRNA targeting (Table S4 in File S1). Taken together, dual sgRNAs targeting at one gene yielded more fragment deletion events.

We assumed a founder rat harbors bi-allelic mutations when the wild type allele was undetectable by PCR genotyping. And for simplicity, we regarded a founder rat carries a monoallelic mutation when the wild type allele was detected. In this study, efficient bi-allelic modifications by the Cas9:sgRNA were observed in 25 potential founders in the *ApoE* gene (Table 1, Fig. 2), 17 potential founders in the *B2m* gene (Table 1, Fig. 2), 11 potential founders in the *Prf1* gene (Table 1, Fig. 2), and 14 potential founders in the *Prkdc* gene (Table 1, Fig. 2). Consequently, potential founder #38 with bi-allelic *ApoE* mutation was sacrificed and observed the level of serum low density lipoprotein (LDL) increased up to 275.5% compared with wild-type control rats (Fig. S3a in File S1); potential founder #36 with bi-allelic *B2m* mutation was sacrificed and showed no B2M expression in the lung (Fig. S3b in File S1); no mature T cell was detected in potential founders #31 and decreased mature B cells with bi-allelic *Prkdc* mutation (Fig. S3c in File S1).

### Off-target analysis

**Figure 4:**
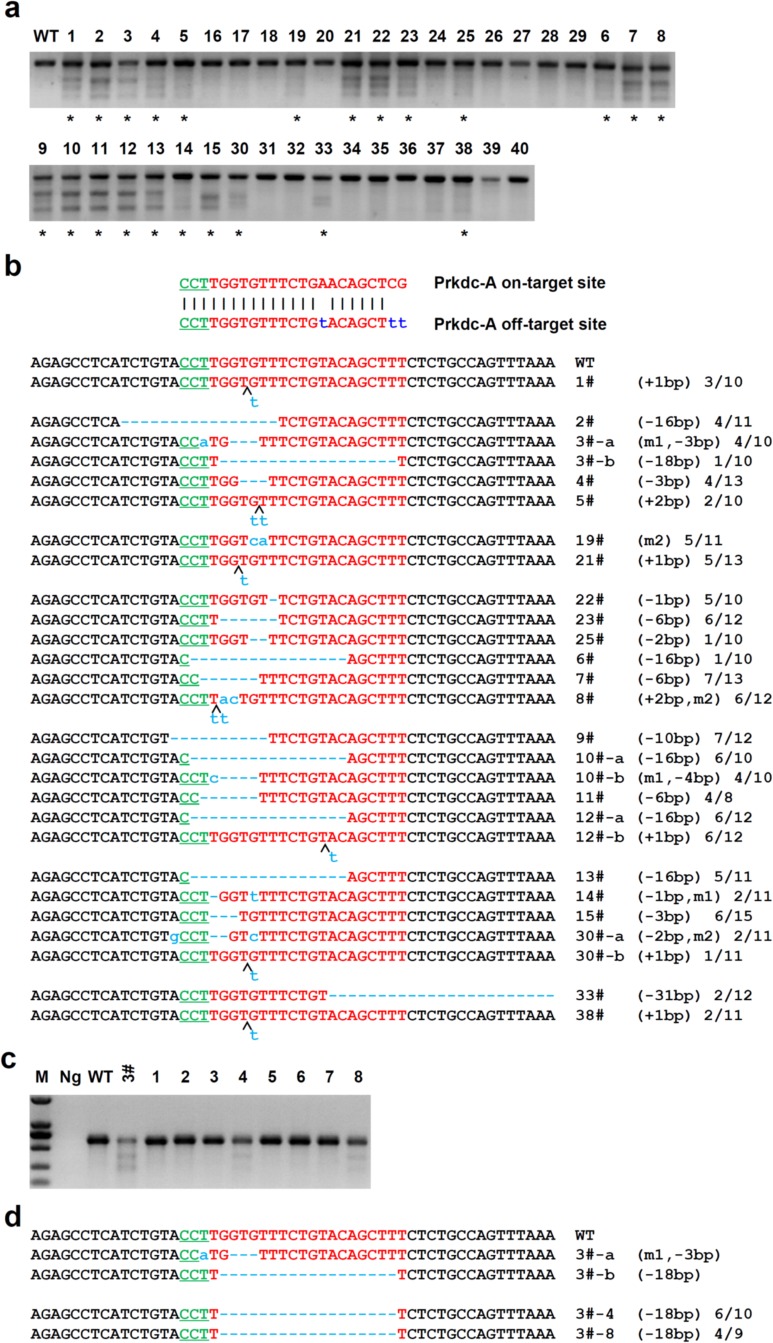
Analysis of the transmission of the off-target mutation. (a) Detection of Cas9:sgRNA-mediated off-target cleavage *of Prkdc* OTS-4 in all founders (#1–40) by T7EN1 cleavage assay. PCR amplicon of *Prkdc* OTS-4 in all 40 founder rats were subjected to T7EN1 cleavage assay as described in methods. Total 23 founders (*) displayed cleavage bands. (b) PCR products with cleavage bands were cloned and sequenced. Sequence result showed OTS-4 indeed mutagenized in the 23 founders. Indels were also detected around 270 bp downstream of the OTS-4 in most colonies, which may be introduced by PCR amplification when Taq encountering repeat sequence. (c) Detection of Cas9:sgRNA-mediated off-target cleavage of *Prkdc* OTS-4 in 8 F1 pups derived from founder #3 by T7EN1 cleavage assay. Mutations were detected in 2 F1 pups (4 and 8). (d) DNA sequences of *Prkdc* OTS-4 in F1 pups 4 and 8. PCR amplicon of the *Prkdc* OTS-4 in founder #3-derived F1 pups 4 and 8 were sequenced. Sequencing result showed one kind of off-target mutation same as the founder #3 was detected in the offspring, indicating that off-target mutation induced by Cas9:sgRNA was heritable.

Recent work suggested that CRISPR-Cas9 can tolerate 1∼3 base pairs mismatch, and then induce off-target-mutation [19]–[21]. Next, we comprehensively investigated off-target damage in mutant rats. We examined 13 potential off-target sites (OTS) for ApoE-A sgRNA, 51 OTS for B2m-A sgRNA, 8 Prf1-A sgRNA and 47 OTS for Prkdc-A sgRNA in 6 selected founders by T7EN1 cleavage assay. Surprisingly, only one real off-target mutation (Prkdc-A OTS-4) was detected from total 119 OTS (Fig. S4 and Table S5 in File S1), demonstrating CRISPR-Cas9 is a reliable rat gene targeting tool. Then we checked Prkdc-A OTS-4 in the other F_0_ rats and found mutations indeed occurred in 23 F_0_ rats by T7EN1 cleavage assay and sequencing (Fig. 4a & 4b). Notably, 8 out of 25 founders from the 5 ng/µl Prkdc-A sgRNA group contained mutations, while all founders (15/15) from the 10 ng/µl Prkdc-A sgRNA group contained mutations, suggesting off-target effect can be minimized by decreasing the concentration of sgRNA.

### Germline transmission analysis and mosaicism

In addition, we determined the transmission of gene modifications by crossing 3 F_0_ mutants (potential founder #3, #19, #26) with wild-type SD rats and determined the genotypes of the F_1_ offspring. The offspring yielded PCR product of same size as potential founders. The PCR products were further analyzed by sequencing. Sequencing results showed the same mutations appeared in offspring as their mutant parent rats (Fig. S5 in File S1), demonstrating that mutations induced by Cas9:sgRNA can be transmitted through germline. Off-target mutation was also determined in founder #3, and the result showed mutation in potential off-target site was transmitted through germline (Fig. 4c). Cas9:sgRNA may continue to induce DNA double strand break (DSB) beyond one-cell stage embryos, resulting in genetic mosaicism. If a mutation cannot be transmitted through germline, or if more than two mutations were detected in the progeny, we assumed the rat had genetic mosaicism. From the germline transmission assay, only one mutation (Fig. S5 in File S1) was not detected in the F_1_ offspring. More than two mutants were detected at *ApoE* (potential founder #10), *B2m* (potential founder #15), *Prkdc* (potential founder #29 and #31) (Figure 2). These results indicate that Cas9:sgRNA also induces genetic mosaicism at low frequency, similar to TALENs. This mosaicism may result from persistent Cas9:sgRNA activity in later embryogenesis, or re-cleavage of certain already alleles [5], [6].

## Discussion

Genetic manipulation of rats is a crucial technique for the study of diseases, especially in the fields of neuroscience, physiology and drug discovery. Generation of precise genetic alterations is limited by rat embryonic stem (ES) cells culture [1]–[2]. In 2009, the gene targeted rat became technically feasible using ZFNs, which can bypass ES cells screening and chimeric rat germline transmission procedure [3]. However, the generation of specific mutant rat is labor intensive by ZFNs or Talens for the need to engineer specific protein pairs for each target site. Recently, another genome editing tool CRISPR/Cas9 has proven to be a simpler way to edit the eukaryotic genome.

Here, we successfully disrupt four genes (*ApoE*, *B2m*, *Prf1*, and *Prkdc*) in one rat in one-step at efficiencies of 24% by co-injection of Cas9 mRNA and sgRNA into one-cell fertilized eggs. During the revision process of this work, two independent studies reported the success of rat genome modifications using CRISPR-Cas9 system [22], [23]. This system eliminates the necessity of engineering specific protein pairs to each target site [5]–[6], [24] and makes it possible to produce mutant rats in a few weeks, which suggests that the CRISPR/Cas9 system is an efficient tool for accelerating the propagation of mutant rats.

Furthermore, rats with mutations in multiple closely linked genes are hard to produce by breeding rats with a single mutation. In this study, we showed that co-injection of Cas9 with multiple sgRNAs can generate rats with multiplex genetic mutations in one step with low off-target effects and mosaicism. In theory, the CRISPR/Cas9 system should enable straightforward generation of rats with multiple mutations in tightly linked genes.

CRISPR/Cas9 system can tolerate sequence mismatch, which may induce off-target-mutation. From our result, only one off-target mutation was detected from total 119 potential targeting sites, demonstrating CRISPR-Cas9 induced off-target-mutation at a very low level. As described above, this system may induce genetic mosaicism. Our results showed that only one rat did not transmit a mutation detected in the tail DNA to its F_1_ offspring. This may be caused by mosaicism or a small sample size. Somatic genetic mosaicism in tail DNA was also at low levels.

Our results confirmed the versatility and reliability of CRISPR/Cas9 system for rat genome editing. It will be interesting to expand this targeting system to produce precise deletions, conditional alleles and insertion of larger DNA fragments to generate knock-in and conditional knock-out rats for the genes of interest.

## Supporting Information

File S1 Figure S1. The pUC57-sgRNA expression vector. The sgRNA expression vector was constructed using the backbone of the pUC57 vector with a Kanamycin resistance gene. The annealed oligos were inserted between the two *Bsa* I restriction sites (blue) downstream of the T7 promoter (red). The construct was linearized by *Dra* I (green) for *in vitro* transcription. Figure S2. Cas9:sgRNA-mediated 4 gene modifications by a mixture of 4 single sgRNAs. (a) PCR identification of sgRNA:Cas9-mediated site-specific cleavage of the endogenous *ApoE*, *B2m*, *Prf1*, and *Prkdc* loci. The genetic modification analysis was performed by PCR amplification of the targeted fragment in the *ApoE*, *B2m*, *Prf1*, and *Prkdc* in 15 potential founder rats (#1∼15) derived from co-microinjection of a mixture of 4 single sgRNAs as described in Table S2 in File S1. Primers used for PCR amplication were described in Table S3 in File S1. Figure S3. Phenotypes of the mutant potential founder rats. (a) Hematobiochemical assay of wild-type control and potential founder #38 harboring bi-allelic *ApoE* mutation. The levels of CHO, TG, HDL, and LDL in serum of founder #38 were quantified. The LDL increased up to 275.5% compared with wild-type control rats. CHO, total cholesterol; TG, triglycerides; HDL, high density lipoprotein; LDL, low density lipoprotein. (b) Western blot analysis of B2M expression in potential founder #36 harboring bi-allelic *B2m* mutations. The expression of B2M in lung of potential founder #36 was not detected by Western blot. (c) Flowcytometry analysis of peripheral blood nucleated cells from wild-type control and founder #31 harboring bi-allelic *Prkdc* mutation. Dot plots represent CD3, CD45RA positive cells for mature T and B cell subpopulations, respectively. Figure S4. Analysis of the off-target effect. Detection of Cas9:sgRNA-mediated off-target mutation in potential founders #25, #7, #8, #30, #39, and #40 by T7EN1 cleavage assay. Marker and wild-type control were located at the left two lanes of the gel. Samples with different pattern of cleavage bands compared with wild-type control were marked with asterisks and sub-cloned for sequencing. Sequence results showed, except OTS-4 of *Prkdc*, all the cleavage activities were induced by single nucleotide polymorphism (SNP), genetic variation (*Prkdc* OTS-37, 27 bp-deletion) or mutations introduced by PCR amplification. Cleavage activities detected in wild-type and potential founders (#) were further confirmed by sequencing. The results showed the mutations were induced by Taq encountering repeat sequence. Figure S5. Analysis of the transmission of the on-target mutation. To analyze the transmission of mutations, potential founders with one (founder #3), two (founder #19), or three (founder #26) mutant genes were selected to cross with wild-type SD rat. (a) Detection of Cas9:sgRNA-mediated on-target cleavage of the endogenous *B2m* in 8 F_1_ pups derived from potential founder #3 by T7EN1 cleavage assay. Mutations were detected in 3 F_1_ pups (1, 4, and 5). (b) DNA sequences of genomic loci in F_1_ pups 1, 4 and 5. PCR amplicon of the targeted fragment at the *B2m* in potential founder #3-derived F_1_ pups 1, 4, and 5 were cloned and sequenced. Sequencing result showed one kind of mutation same as the founder #3 was detected in the offspring, indicating that mutations induced by Cas9:sgRNA was transmittable. However, 351 bp-deletion mutants can't be detected in offspring, suggesting that Cas9 function may not only in one-cell, but also in the later stage. (c) Detection of Cas9:sgRNA-mediated on-target cleavage of the endogenous *B2m* and *Prkdc* in 12 F_1_ pups derived from potential founder #19 by T7EN1 cleavage assay. The mutations were detected in all 12 F_1_ pups. (d) DNA sequences of genomic loci in mutant pups. PCR amplicon of the targeted fragment at the *B2m* and *Prkdc* in potential founder #19-derived F_1_ pups were cloned and sequenced. Sequencing result showed the mutations same as the founder #19 were detected in the offspring. Two kinds of mutation of *Prkdc* were all transmittable, indicating mosaicism induced by Cas9:sgRNA. (e) Detection of Cas9:sgRNA-mediated on-target cleavage of the endogenous *ApoE* by PCR, *B2m* and *Prkdc* by T7EN1 cleavage assay in 10 F_1_ pups derived from potential founder #26. The mutations were detected in F1 pups. (f) DNA sequences of genomic loci in mutant pups. Smaller band of PCR amplicon of *ApoE* were gel extracted and sequenced. PCR amplicon of the targeted fragment at the *B2m* and *Prkdc* in potential founder #26-derived F1 pups were cloned and sequenced. Sequencing result showed the mutations same as the founder #26 were detected in the offspring. Table S1. Oligonucleotides for generating sgRNA expression vectors. Table S2. Summary of embryo injections of sgRNA:Cas9. Table S3. Primers for amplifying sgRNA targeted loci. Table S4. Summary of mutations of multiple genes. Table S5. Summary of the alleles for putative off-target sites. Table S6. Primers for amplifying off-target sites.
(ZIP)
