# Supplementary material for: Heritable Multiplex Genetic Engineering in Rats Using CRISPR/Cas9
Source: PLoS One. 2014 Mar 5;9(3):e89413. doi: 10.1371/journal.pone.0089413 (PMC3943732; doi:10.1371/journal.pone.0089413)
Supplement: File S1 — Figure S1. The pUC57-sgRNA expression vector. The sgRNA expression vector was constructed using the backbone of the pUC57 vector with a Kanamycin resistance gene. The annealed oligos were inserted between the two Bsa I restriction sites (blue) downstream of the T7 promoter (red). The construct was linearized by Dra I (green) for in vitro transcription. Figure S2. Cas9:sgRNA-mediated 4 gene modifications by a mixture of 4 single sgRNAs. (a) PCR identification of sgRNA:Cas9-mediated site-specific cleavage of the endogenous ApoE, B2m, Prf1, and Prkdc loci. The genetic modification analysis was performed by PCR amplification of the targeted fragment in the ApoE, B2m, Prf1, and Prkdc in 15 potential founder rats (#1∼15) derived from co-microinjection of a mixture of 4 single sgRNAs as described in Table S2 in File S1. Primers used for PCR amplication were described in Table S3 in File S1. Figure S3. Phenotypes of the mutant potential founder rats. (a) Hematobiochemical assay of wild-type control and potential founder #38 harboring bi-allelic ApoE mutation. The levels of CHO, TG, HDL, and LDL in serum of founder #38 were quantified. The LDL increased up to 275.5% compared with wild-type control rats. CHO, total cholesterol; TG, triglycerides; HDL, high density lipoprotein; LDL, low density lipoprotein. (b) Western blot analysis of B2M expression in potential founder #36 harboring bi-allelic B2m mutations. The expression of B2M in lung of potential founder #36 was not detected by Western blot. (c) Flowcytometry analysis of peripheral blood nucleated cells from wild-type control and founder #31 harboring bi-allelic Prkdc mutation. Dot plots represent CD3, CD45RA positive cells for mature T and B cell subpopulations, respectively. Figure S4. Analysis of the off-target effect. Detection of Cas9:sgRNA-mediated off-target mutation in potential founders #25, #7, #8, #30, #39, and #40 by T7EN1 cleavage assay. Marker and wild-type control were located at the left two lanes of the [file pone.0089413.s001.zip › File S1/Table S3.pdf]

Name	Sequence (5’-3’)	Amplicon
ApoE-NS1	GGATTGATGCTCAGAGGGTAAAGGT	699 bp
ApoE-NAS1	CATCCCAGTATCCAGAAGGCTAAAG	
ApoE-NS2	GGGAGACAAACTAAGATCGTGAGGTAAC	2435 bp
ApoE-NAS2	GCCTGTATCTTCTCCATTAGGTTTGC	
B2M-S1	AGAAGACAGTGCTAAACGCCAGG	607 bp
B2M-AS1	TCGCTAACAGTCTCAGAATGCAG	
B2M-S2	GACTGTGATCTCTGAACCGGGAAG	2759 bp
B2M-AS2	CACATTCCCTCATGCTGTCCTTC	
Prf1-S1	TGCTACACTGCCACTCGGTCAG	535 bp
Prf1-AS1	CTAGACCACTTAGAGTGTCCCAC	
Prkdc-S1	AGATGCACTTGGGTAAACACCTG	609 bp
Prkdc-AS1	GTAAGTGACAGGTATCTCACCAG	
